# Phytochemical Profile and Biological Properties of *Colchicum triphyllum* (Meadow Saffron)

**DOI:** 10.3390/foods9040457

**Published:** 2020-04-08

**Authors:** Biancamaria Senizza, Gabriele Rocchetti, Murat Ali Okur, Gokhan Zengin, Evren Yıldıztugay, Gunes Ak, Domenico Montesano, Luigi Lucini

**Affiliations:** 1 Department for Sustainable Food Process, Università Cattolica del Sacro Cuore, Via Emilia Parmense 84, 29122 Piacenza, Italy; biancamaria.senizza@unicatt.it (B.S.); luigi.lucini@unicatt.it (L.L.); 2 Department of Biology, Science Faculty, Selcuk University, Campus, 4213 Konya, Turkey; murataliokur@hotmail.com (M.A.O.); gokhanzengin@selcuk.edu.tr (G.Z.); akguneselcuk@gmail.com (G.A.); 3 Department of Biotechnology Science Faculty, Selcuk University, Campus, 4213 Konya, Turkey; eytugay@gmail.com; 4 Department of Pharmaceutical Sciences, Food Science and Nutrition Section, University of Perugia, Via S. Costanzo 1, 06126 Perugia, Italy

**Keywords:** meadow saffron, metabolomics, UHPLC-QTOF-mass spectrometry, extraction methods, bioactive compounds, antioxidants

## Abstract

In this work, the phytochemical profile and the biological properties of *Colchicum triphyllum* (an unexplored Turkish cultivar belonging to Colchicaceae) have been comprehensively investigated for the first time. Herein, we focused on the evaluation of the in vitro antioxidant and enzyme inhibitory effects of flower, tuber, and leaf extracts, obtained using different extraction methods, namely maceration (both aqueous and methanolic), infusion, and Soxhlet. Besides, the complete phenolic and alkaloid untargeted metabolomic profiling of the different extracts was investigated. In this regard, ultra-high-performance liquid chromatography coupled with quadrupole time-of-flight mass spectrometry (UHPLC-QTOF-MS) allowed us to putatively annotate 285 compounds when considering the different matrix extracts, including mainly alkaloids, flavonoids, lignans, phenolic acids, and tyrosol equivalents. The most abundant polyphenols were flavonoids (119 compounds), while colchicine, demecolcine, and lumicolchicine isomers were some of the most widespread alkaloids in each extract analyzed. In addition, our findings showed that *C. triphyllum* tuber extracts were a superior source of both total alkaloids and total polyphenols, being on average 2.89 and 10.41 mg/g, respectively. Multivariate statistics following metabolomics allowed for the detection of those compounds most affected by the different extraction methods. Overall, *C. triphyllum leaf* extracts showed a strong in vitro antioxidant capacity, in terms of cupric reducing antioxidant power (CUPRAC; on average 96.45 mg Trolox Equivalents (TE)/g) and ferric reducing antioxidant power (FRAP) reducing power (on average 66.86 mg TE/g). Interestingly, each *C. triphyllum* methanolic extract analyzed (i.e., from tuber, leaf, and flower) was active against the tyrosinase in terms of inhibition, recording the higher values for methanolic macerated leaves (i.e., 125.78 mg kojic acid equivalent (KAE)/g). On the other hand, moderate inhibitory activities were observed against AChE and α-amylase. Strong correlations (*p* < 0.01) were also observed between the phytochemical profiles and the biological activities determined. Therefore, our findings highlighted, for the first time, the potential of *C. triphhyllum* extracts in food and pharmaceutical applications.

## 1. Introduction

*Colchicum triphyllum* Kunze is a spring/autumn-flowering species belonging to the Colchicaceae family, widely distributed in Turkey and Balkans [1]. It is also known as “autumn crocus” or “meadow saffron”. Plants belonging to Colchicaceae are mainly used in pharmaceutical applications, thanks to therapeutic, anti-inflammatory, and antitumoral activities [2] attributed to the presence of colchicinoids (alkaloids), such as colchicine and demecolcine. In this regard, colchicine is used in the treatment of gout [3] and Behcet’s disease [4], while demecolcine, together with trimethyl-colchicine acid methyl ester, demonstrated anti-neoplastic activity and is particularly suitable for the treatment of leukemia [5]. Besides, bioactive compounds characterizing plants belonging to the Colchicaceae family, such as alkaloids (e.g., colchicine), have been characterized and have been widely studied because of their beneficial effects for the treatment of cirrhosis, psoriasis, and amyloidosis [6]. Interestingly, less toxic derivatives of colchicine have also been studied as anticancer and antitumoral agents. *Colchicum* spp. also contains a considerable distribution of bioactive compounds, such as polyphenols. In particular, according to the literature [7], the most abundant (poly)-phenolic compounds are lignans, flavonoids, phenolic acids, and tannins. 

Notably, *Colchicum* leaves share morphological similarities (mainly looking at leaves) with other plant species, such as *Allium ursinum* L. (wild garlic); fortunately, poisoning is rare, although some accidents (with also lethal outcomes) caused by the ingestion of toxic *Colchicum* alkaloids have been described in the scientific literature [8]. In addition, some *Colchicum* species (mainly *C. autumnale* L.) may be confused for the same reasons as Crocus spp. (mainly *Crocus sativus*); despite their morphological similarity, *Colchicum* flowers are typically larger with six stamens, while Crocus flowers are smaller with three longer stamens. Another issue related to saffron (*Crocus sativus*) is the lack of knowledge by consumers about the correct shape and botanical characteristics of the products, thus leading to potential episodes of adulteration and counterfeiting procedures on the market. In fact, besides morphological analogies, some plant-tissues from *Colchicum* spp. are extremely toxic, thus potentially affecting human health.

There are previous works based on the description and characterization of *Colchicum* spp. and its alkaloid distribution (focused mainly on colchicine and demecolcine) in several Jordanian *Colchicum* species. Recently, Rocchetti and co-authors [9] profiled, for the first time, flowers, leaves, and tubers of *Colchicum szovitsii* subsp. *szovitsii*, showing a great abundance of flavonols, phenolic acids, and total alkaloids that were the main components responsible for biological activities detected. However, to the best of our knowledge, there are no comprehensive studies based on a detailed characterization of both polyphenols and alkaloids characterizing different parts (i.e., flower, leaves, tuber) of *C. triphyllum* and based on untargeted metabolomics (i.e., ultra-high-performance liquid chromatography coupled with quadrupole time-of-flight (UHPLC-QTOF) mass spectrometry). Besides, considering that, to date, no efficient methods to synthesize *Colchicum* alkaloids have been found; colchicine and other alkaloids are mainly obtained from plant sources by different extractions techniques. Therefore, in this work, infusion, maceration (using methanol and water) together with Soxhlet extraction techniques were used to promote the extraction of both polyphenols and alkaloids from *C. triphyllum*, aiming to identify the most discriminant markers of each extraction technique used. Finally, in vitro antioxidant and enzyme inhibitory assessments were carried out to investigate the biological and pharmaceutical potential of this plant species. 

## 2. Materials and Methods 

### 2.1. Plant Material

The plant materials of *Colchicum triphyllum,* namely flowers, leaves, and tubers, were collected at Konya in Turkey in 2019 (Konya, around Silla Dam Lake, steppes 1200 m; Collection date: 03.02.2019). The plant materials were collected and identified by botanist Dr. Evren Yildiztugay (Selcuk University, Department of Biotechnology, Konya, Turkey, Voucher number: EY-2968). In the sampling, about twenty plants were collected in the same population. The plant materials were cleaned (first, washing with tap water and then rising by distilled water), and soil and other contaminants were removed. The plant parts, namely flowers, leaves, and tubers, were carefully separated, and these plants were dried in a shaded and well-ventilated environment at the Department of Biology, Selcuk University. After drying (about 10 days), the plant materials were powdered by using a laboratory mill (Retsch, SM-200), and the powdered materials were used to obtain extracts in the same week. The powdered plant materials were stored in well-ventilated conditions in the dark (about 20 ˚C). We performed the analysis in about one month after sampling. 

### 2.2. Extraction Methods

In this work, three different extraction methods using different solvents were tested. In this regard, to obtain extracts, we performed infusion, maceration, and Soxhlet extraction techniques. Regarding infusion, the plant materials (5 g) were kept in 100 mL of boiling water for 20 min and then filtered. In the maceration technique, the plant materials (5 g) were mixed with 100 mL of both methanol and water for 24 h at room temperature. In the Soxhlet technique, the plant materials (5 g) were extracted with 100 mL methanol by using a Soxhlet apparatus for 6 h. Final extracts were obtained by using a vacuum evaporator and lyophilization. Finally, each obtained extract was stored in a refrigerator until further analyses. 

### 2.3. UHPLC-QTOF Profiling of Polyphenols and Alkaloids

The untargeted phytochemical profile of the different *C. triphyllum* extracts was investigated through ultra-high-pressure liquid chromatography (Agilent 1290 HPLC liquid chromatograph; Agilent Technologies, Santa Clara, CA, USA) coupled to a quadrupole-time-of-flight mass spectrometer (Agilent 6550 iFunnel; Agilent Technologies, Santa Clara, CA, USA). The experimental conditions for the analysis of plant extracts using untargeted metabolomics were optimized in previous works from our research group [9,10,11]. The mass spectrometer acquired ions in the range 50–1200 m/z in positive (ESI+) scan mode. Three technical replications were considered, with an injection volume of 6 µL. An in-house database built, combining Phenol-Explorer 3.6 with some of the most important alkaloids reported in the literature on Colchicaceae, was then used for annotation purposes, exploiting the entire isotopic profile (i.e., combining monoisotopic accurate mass, isotopic ratios, and spacing) with a mass accuracy below 5 ppm. Therefore, the approach used was based on a Level 2 of identification (i.e., putatively annotated compounds), as set out by the COSMOS Metabolomics Standards Initiative [12,13,14]. Afterward, Agilent Profinder B.06 software was used for post-acquisition data filtering, retaining only those compounds identified within 100% of replications in at least one condition. Thereafter, to provide more quantitative information on the different annotated compounds, polyphenols were first ascribed into classes and subclasses and then quantified using standard solutions (80/20, *v*/*v* methanol/water) of pure standard compounds analyzed with the same method [9]. The following phenolic classes were targeted: anthocyanins (quantified as cyanidin equivalents), flavones (quantified as luteolin equivalents), flavonols (quantified as catechin equivalents), lignans (quantified as sesamin equivalents), low-molecular-weight phenolics (quantified as tyrosol equivalents), and phenolic acids (quantified as ferulic acid equivalents). Finally, a calibration curve of sanguinarine (Sigma grade, Sigma–Aldrich, S. Louis, MO, USA) was used to estimate the total alkaloid content. The results were finally expressed as mg equivalents/g dry matter.

### 2.4. In Vitro Antioxidant Capacity and Inhibitory Potential

For in vitro antioxidant capacity, different test systems, including radical quenching, reducing power, phosphomolybdenum, and ferrous ion chelating, were employed. The details of the methods are described in our earlier papers [15,16]. The results were reported as mg Trolox Equivalents (TE)/g extract and ethylenediaminetetraacetic acid (EDTA) equivalents (for ferrous ion chelating; mg EDTAE/g extract). For enzyme inhibitory activities, key enzymes for global health problems were selected, namely, α-amylase and α-glucosidase, acetylcholinesterase (AChE), butyrylcholinesterase (BChE), and tyrosinase and the inhibitory activities were compared to standard drugs (acarbose for amylase and glucosidase; galantamine for AChE and BChE; kojic acid for tyrosinase). All assays were performed considering three technical replications. 

### 2.5. Statistical Analysis and Chemometrics

A one-way analysis of the variance (ANOVA) was performed considering data from each assay and using the software PASW Statistics 26.0 (SPSS Inc., Chicago, IL. USA), followed by a Duncan’s post hoc test (*p* > 0.05). Pearson’s correlations (*p* < 0.05; two-tailed) were also calculated using PASW Statistics 26.0. Afterward, the metabolomics-based dataset exported from Mass Profiler Profession B.12.06 (Agilent Technologies, Santa Clara, CA, USA) was elaborated into a second software, namely SIMCA 13 (Umetrics, Malmo, Sweden) for supervised orthogonal projections to latent structures discriminant analysis (OPLS-DA), as previously reported in previous works from our research group [9]. Two OPLS-DA models were built; the first one highlighted the differences in the phytochemical profiles as imposed by the extraction methods, while the second model showed the differences between the three plant-organs under investigation. Finally, the variables selection method VIP (i.e., variables’ importance in projection) was used to evaluate those compounds mostly affected by the different extraction methods, together with those better discriminating the plant-organs. In particular, polyphenols and alkaloids showing a VIP score > 1 have been considered as marker compounds.

## 3. Results and Discussion

### 3.1. Phytochemical Profiling of the Different Extracts

To characterize the polyphenol and alkaloid composition of the different *Colchicum triphyllum* extracts we used untargeted metabolomics based on UHPLC-QTOF mass spectrometry. According to this approach, 285 compounds were putatively identified in the different matrix extracts, mainly including alkaloids, flavonoids (such as anthocyanins, flavonols, and flavones), lignans, and low-molecular-weight phenolic acids. Each compound annotated is provided in Supplementary Table S1 together with its abundance and composite mass spectra. Overall, the most abundant compounds detected when considering the metabolomic dataset were alkaloids (such as colchicine, demecolcine, and y-lumicolchicine), anthocyanins (such as petunidin 3-*O*-rutinoside and cyanidin 3-*O*-sophoroside), flavones (mainly apigenin and luteolin glucosides), and flavonols (mainly isomeric forms of kaempferol and quercetin). Regarding the class of lignans, the most abundant annotated compounds were secoisolariciresinol, pinoresinol, and its isomer, matairesinol (Supplementary Table S1). Afterward, a semi-quantitative approach based on standard compounds was used to evaluate the concentration of phytochemicals in the studied plant matrices. The results of this semi-quantitative approach are reported in Table 1.

As can be observed, the three plant matrices were predominantly rich (*p* < 0.05) in alkaloids and lignans when compared to the other classes of compounds, while flavonols and anthocyanins showed the lower (*p* < 0.05) concentration; it is also interesting to notice that, when considering *C. triphyllum* tuber extracts, neither anthocyanins nor flavonols were detected. Besides, looking at *C. triphyllum* flower extracts, the maceration-water extraction method was found to promote the highest recovery of lignans and alkaloids (3.01 and 2.08 mg/g, respectively), while maceration-MeOH extraction encourages the recovery of anthocyanins, flavonols, phenolic acids, and tyrosols. Interestingly, Soxhlet extraction promoted the highest recovery of flavones (2.52 mg/g). *Colchicum* flowers are reported to be very similar from a morphological point of view to those of *Crocus Sativus L*. Overall, both plant species can be considered as a good source of (poly)-phenolic compounds In this regard, the saffron flower has been described as rich in flavonoids (such as flavonols and flavones), hydroxycinnamic acids, and lignans [10,17,18]. Regarding saffron alkaloids, Amin Mir and co-authors [19] found this class of compounds in both water and methanolic extracts of flowers. Another study by Hosseinzadeh et al. [20] based on the phytochemical screening of different *Crocus* extracts, highlighted the distribution of flavonoids (including anthocyanins) and tannins in both aqueous and ethanolic petal extracts, while alkaloids and saponins characterized the aqueous and ethanolic stigmas extracts. However, our data are difficult to compare with previously cited works, considering that in this work, both polyphenols and alkaloids were evaluated, targeting the whole flower. Recently, Jadouali and co-authors [21] evaluated the total phenolic content of different flower parts of Moroccan *Crocus sativus* L., showing a value of 54.59 mg gallic acid equivalents (GAE)/g for the whole saffron flower. 

Considering the lack in the literature of similar comprehensive phytochemical screening on *C. triphyllum* extracts, we compared our findings with a previous work focused on a different species, namely *C. szovitsii*. In particular, the most abundant polyphenols characterizing *C. szovitsii* plant extracts were flavonols, phenolic acids, and tyrosols equivalents [9], while anthocyanins and flavanols were found to be less abundant. Another interesting result was obtained when comparing the extraction efficiency of polyphenols between the two *Colchicum* species and using Soxhlet-MeOH extractions. In particular, Soxhlet-MeOH promoted a better extraction of flavonols and flavones in tubers of *C. szovitsii* when compared to *C. triphyllum*, being 1.58 and 0.81 mg/g vs. not detectable values and 0.45 mg/g, respectively. Interestingly, the same extraction method (i.e., Soxhlet-MeOH) promoted a better recovery of lignans and tyrosols in *C. tryphillum*, being 10.60 and 2.85 mg/g, respectively. Regarding the alkaloids putatively annotated in *C. triphyllum* extracts, tubers obtained by water maceration showed the highest content (3.46 mg/g). This aspect is worthy of interest, considering that the colchicinoids (mainly colchicine and derivatives) are among the highly poisonous water-soluble alkaloids detected in flowers and seeds of *Colchicum* genus [22]. Regarding *C. szovitsii* leaf extracts, flavonols were better extracted by exploiting Soxhlet (21.95 mg/g), while phenolic acids and tyrosol equivalents by infusion (3.52 and 3.68 mg/g, respectively). On the other hand, *C. triphyllum* was revealed to be a great source of lignans (possessing a potential estrogenic activity), recording an average value of 3.63 mg/g when considering all the extraction methods tested (Table 1). Finally, concerning the total alkaloid content, *C. triphyllum* and *C. szovitsii* leaf extracts showed a comparable content, being 2.79 and 2.65 mg/g when considering Soxhlet and water-maceration, respectively. 

### 3.2. Multivariate Statistical Discrimination of the Different Extraction Methods

**Figure 1:**
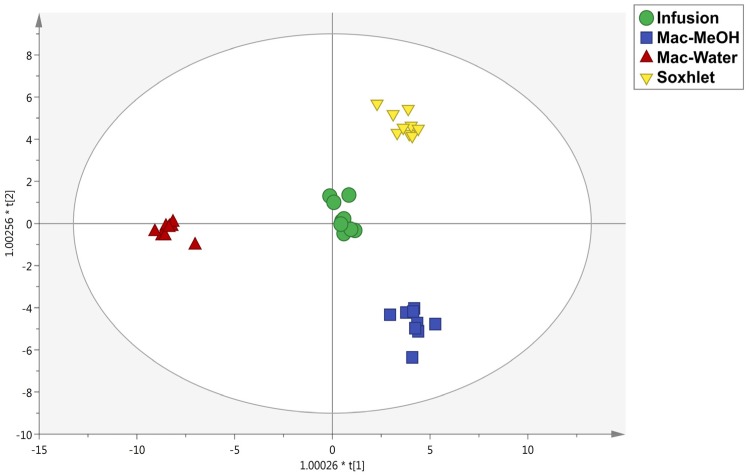
Orthogonal projections to latent structures discriminant analysis (OPLS-DA) score plot built according to polyphenol and alkaloid profiling and considering the different extraction methods as class membership criteria.

Considering specifically the extraction type, a supervised multivariate statistical approach was carried out to find the compounds allowing the discrimination between the different methods. In more detail, an OPLS-DA (orthogonal projection to latent structures discriminant analysis) was depicted, followed by the variables importance in projection (VIP) method, to select those compounds mostly affected by the different extraction methods. As can be observed in Figure 1, a clear separation based on alkaloids and polyphenols content was achieved. In particular, each extraction method provided a differential phytochemical profile, although water-maceration extracts were different from the others. The model parameters were more than acceptable, being R^2^Y (cum) (goodness-of-fit) = 0.97 and Q^2^ (cum) (goodness-of-prediction) = 0.91. Besides, the model was cross-validated and showed neither suspect nor strong outliers. Afterward, the variables selection method VIP was exploited to find those metabolites mostly influenced by the extraction method used. Hence, 26 compounds were those possessing a VIP score > 1.2, including flavonoids (7 compounds), followed by low-molecular-weight phenolics (7 compounds), phenolic acids (6 compounds), and lignans (5 compounds). The list containing the remaining VIP compounds (1.2 < VIP score < 1) is reported in Supplementary Table S1. Interestingly, only one alkaloid, namely colchiceine, possessed a VIP score > 1.2 (i.e., 1.27). Regarding polyphenols, the higher VIP score was recorded for the lignan 1-acetoxypinoresinol, mainly found in tubers and leaves but not in flowers, followed by the caffeoylquinic acid isomers (VIP score = 1.39), whose presence was only detected in flowers treated with mac-MeOH. Furthermore, the lignans sesaminol, sesamolin, and episesaminol (VIP score = 1.36) were also better extracted with the infusion method. Some studies established that ethanol and methanol plant and fruit extracts can provide a better recovery of polyphenols when compared to water as the solvent, but the efficiency is strictly related to the extraction time [20]. Moreover, changes in temperature and the solvent-mixtures chosen could enhance and/or reduce the extraction efficiency. The Soxhlet method is a well-established technique requiring a smaller quantity of solvent compared to maceration, but according to literature, it is not suggested to promote the extraction of thermolabile compounds [23].

**Figure 2:**
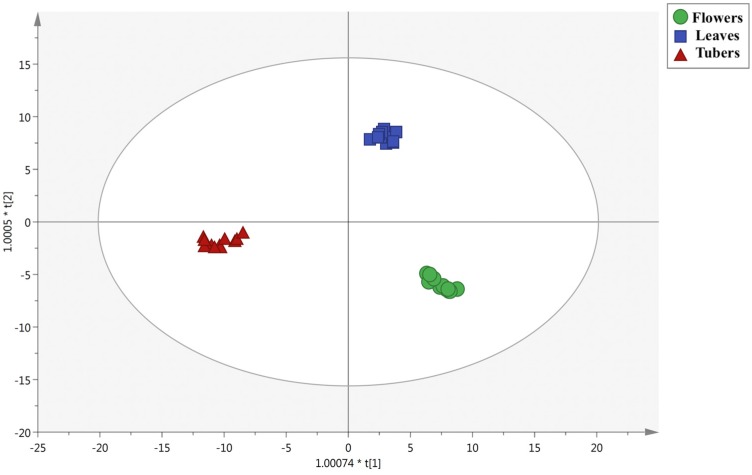
Orthogonal projections to latent structures discriminant analysis (OPLS-DA) score plot built according to polyphenol and alkaloid profiling and considering the different plant organs (i.e., flowers, leaves, and tubers) as class membership criteria.

Thereafter, a second OPLS-DA model was built to check the major differences between the organs under investigation. The OPLS-DA score plot is reported in Figure 2. 

As can be observed from Figure 2, the second latent vector t[2] provided a clear discrimination between tubers and the other *C. triphyllum* extracts, while the first latent vector t[1] revealed a more exclusive phytochemical profile for the leaf extracts. The goodness of the prediction model built on phenolics and alkaloids was confirmed by inspecting the goodness of fit and prediction, being 0.97 and 0.91, respectively. Finally, to check those compounds allowing the discrimination of the different organs, the VIP method was exploited, and the VIP markers can be found in Supplementary Table S1. Overall, 78 compounds were characterized by a VIP score > 1, being 34 flavonoids, 17 alkaloids, 11 phenolic acids, 11 lower-molecular-weight phenolics, and 5 lignans. In particular, the highest VIP scores were recorded for three alkaloids, namely androbine (1.51), autumnaline (1.46), and colchicoside (1.42). Looking at polyphenols, the most discriminant compounds highlighted by the VIP selection method were genistin (1.39), phlorin (1.35), and pelargonidin 3-*O*-glucoside (1.34). Overall, androbine was a specific marker of leaf extracts; autumnaline was detected in both tubers and leaves), while colchicoside was a marker of both tubers and flowers. Regarding polyphenols, genistin and pelargonidin 3-*O*-glucoside were detected mainly in flower extracts, while phlorin was a specific marker detected in tubers (Supplementary Table S1). Overall, our findings revealed a higher discrimination potential of alkaloids when compared to polyphenols. To date, more than 150 structurally elucidated alkaloids have been described for Colchicaceae genera [24]. In particular, eight distinct structural types of alkaloids characterize the Colchicaceae family, being phenethylisoquinolines (e.g., autumnaline), homoproaporphines (e.g., jolantine), homoaporphines (e.g., merobustine), androcymbines (e.g., szovitsidine), colchicines (e.g., colchicine), allocolchicines (e.g., jerusalemine), lumicolchicines (e.g., γ-lumicolchicine), and homoerythrinans (e.g., taxodine). Therefore, our findings (Supplementary Table S1) are completely following with the typical alkaloid composition reported for Colchicaceae plants. Regarding the polyphenols annotated, few comprehensive works based on high-resolution mass spectrometry are available in the literature. In previous work, Toplan et al. [1] reviewed the importance of *Colchicum* species in modern therapy together with its significance in Turkey, showing that these plants are very abundant in alkaloids, followed by polyphenols (mainly phenolic acids and flavonoids). However, looking to the different *Colchicum* species, few compounds are reported, namely ferulic acid, vanillin, luteolin, coumaric acid, caffeic acid, and 3,4-dihiydroxibenzaldehyde. Therefore, further works are required to better explore and give more insight on the phenolic composition of different *Colchicum* species. 

### 3.3. In Vitro Antioxidant Capacity of the Tested Extracts

To date, the scientific evidence on the biological and pharmacological activities of extracts and bioactive compounds deriving from plants is very strong [25,26,27,28,29,30,31]. In the last years, many studies have explored the association between the ingestion of bioactive compounds and decreased risk of non-communicable diseases. Besides, considering that many non-nutrients with putative health benefits are reducing agents, hydrogen donors, singlet oxygen quenchers, or metal chelators, measurement of antioxidant activity using in vitro assays have become very popular over recent decades. In the current study, we investigated the antioxidant capacities of *C. triphyllum* flower, tuber, and leaf extracts by measuring their total in vitro antioxidant capacity (phosphomolybdenum assay), DPPH (2,2-diphenyl-1-picrylhydrazyl) and [2,2′-azinobis-(3-ethylbenzothiazoline-6-sulfonate)] (ABTS) scavenging capacity, ferric and cupric reduction activity (FRAP and CUPRAC), and metal chelating activity. However, as widely suggested in the recent scientific literature [32], these colorimetric and in vitro methods present many pitfalls and should be used as screening tools, thus supported by a comprehensive LC-MS characterization and quantification of those antioxidant compounds likely responsible of the activity observed. Based on the experimental findings (Table 2), it was observed that all of the studied extracts were characterized by potential health-promoting properties. Radical scavengers can prevent free radical-induced macromolecules or tissue damage by directly neutralizing free radicals and accepting or donating electron(s) to eliminate the unpaired condition of the radical [33]. Among the different *C. triphyllum* extracts tested in this study, we found that leaf extracts were the most active DPPH scavengers, recording an average value (when considering all the extraction method) of 45.22 mg TE/g. On the other hand, the methanolic tuber extracts obtained using the maceration method showed a similar activity, being 46.17 mg TE/g (Table 2). Regarding ABTS assay, the maceration method (based on methanol as extraction solvent) was found to promote the highest activity in tubers and leaves (being 50.93 and 52.55 mg TE/g, respectively). Overall, intriguing differences were outlined in the different organ-specific extracts when considering the cupric reduction activity (CUPRAC). In this regard, *C. triphyllum* leaf methanolic extracts showed higher values than flowers and tubers, recording 109.63 mg TE/g (using maceration method) and 123.34 mg TE/g (using Soxhlet method). Finally, regarding the other assays, FRAP ranged from 25.02 (infused flowers) up to 70.80 mg TE/g (leaves extracted using Soxhlet), metal chelating from 1.89 (tubers extracted using Soxhlet) up to 33.88 mg EDTAE/g (infused tubers), and total in vitro antioxidant capacity (phosphomolybdenum method) from 0.33 (infused tubers) up to 1.52 mmol TE/g (water macerated leaves). Therefore, taken together, our findings demonstrate that *C. triphyllum* leaf extracts were the most active as radical scavengers. This finding was in line with a previous work [9] focusing on another novel cultivar from Turkey, namely *Colchicum szovitsii subsp. szovitsii*.

### 3.4. Enzyme Inhibitory Activity of the Tested Extracts 

To investigate the enzyme inhibitory capacity of the different *C. triphyllum* extracts, the cholinesterases AChE and BChE, together with the enzymes tyrosinase, α-amylase, and α-glucosidase were considered. The results obtained using the above-mentioned enzymes are shown in Table 3. 

According to literature, the enzyme cholinesterase is a significant therapeutic target to alleviate the deterioration of cholinergic neurons in the brain and the loss of neurotransmission, i.e., one of the major causes of Alzheimer’s disease [34]. Overall, AChE inhibition activity was detected in each *C. triphyllum* leaf extract analyzed (Table 3). In particular, the highest activity values were recorded for tuber extracts (obtained by maceration-MeOH) and leaf extracts (obtained through Soxhlet-MeOH), being 4.80 and 4.56 mg galatamine equivalent (GALAE)/g, respectively. No activity against AchE was observed when considering infusion and maceration-water techniques for flowers and tubers extracts. Considering BChE, only methanolic maceration (3.91 mg GALAE/g) and Soxhlet-MeOH of *C. triphyllum* tubers (7.42 mg GALAE/g) were able to produce effective inhibitory activity against this enzyme. Regarding the enzyme tyrosinase, it plays an important role in the melanogenesis and enzymatic browning, so its inhibitors can be considered as anti-browning compounds in food and agriculture industries and as depigmentation agents in the cosmetic and medicinal industries [35]. In our experimental conditions, we found that both Soxhlet and maceration (using methanol as extraction solvent) were able to produce extracts characterized by a strong tyrosinase inhibitory ability. In particular, the highest activity was recorded for *C. triphyllum* leaves using methanolic maceration (i.e., 125.78 mg KAE/g). Finally, regarding two of the most important enzymes from a nutritional standpoint, namely α-amylase and α-glucosidase, we found a low α-amylase inhibition potential for all the tested extracts, from 0.13 (for infused tubers) up to 0.73 mmol acarbose equivalent (ACAE)/g (for leaves and tubers, using Soxhlet and methanolic maceration, respectively). Interestingly, the α-glucosidase inhibition activity was observed only for flower and leaf extracts, with the maximum average activity recorded for leaves (i.e., 1.37 mmol ACAE/g). These two enzymes are potential targets in producing lead compounds for the management of diabetes. Therefore, further research on *C. triphyllum* extracts could open a new perspective for the management of health-related and metabolic problems. 

Regarding a possible comparison between different *Colchicum* species, there are few works available in the literature to make a realistic discussion. That is why we decided to compare our findings with a previous work focused on the chemical characterization of *Colchicum szovitsii* [9]. Overall, the authors showed that *C. szovitsii* possessed a strong tyrosinase inhibitory action, mainly when considering methanolic macerated leaf extracts (i.e., 116.92 mg KAE/g), thus confirming our findings. Similar trends were observed for the remaining tested enzymes. Interestingly, when considering the existing literature on saffron (*Crocus sativus*), similar activities have been reported on different saffron extracts [36], with crocetin, dimethylcrocetin, and safranal the mainly responsible for acetylcholinesterase inhibitions, as revealed by both molecular docking and in vitro enzymatic studies. Besides, Menghini and co-authors [37] showed that saffron stigmas are characterized by both AChE and BChE inhibitions (i.e., 2.51 and 3.44 mg GALAE/g, respectively) and inhibition towards amylolytic enzymes, such as α-amylase (0.44 mmol ACAE/g) and α-glucosidase (6.34 mmol ACAE/g). The same authors also showed that these values are lower when considering the combination of *C. sativus* tepals and anthers. 

### 3.5. Correlations

Pearson’s correlation coefficients were then calculated to check the contribution of polyphenols and alkaloids to the antioxidant and enzymatic related properties observed when considering the different *C. triphyllum* extracts. A summarizing table reporting all of the correlation coefficients for each part analyzed (i.e., flowers, tubers, and leaves) is reported in Supplementary Table S1. Overall, a positive and significant (*p* < 0.05) correlation coefficient was found between total alkaloids and in vitro total antioxidant capacity (phosphomolybdenum method) when considering *C. triphyllum* tuber extracts, with a correlation coefficient of 0.673. Besides, no significant correlations coefficients were recorded for leaves, flowers, and tubers when considering total alkaloids and enzymatic assays. Regarding polyphenols, we found significant correlations mainly between anthocyanins characterizing *C. triphyllum* flower extracts and enzymatic inhibitory assays. In particular, the highest correlation coefficient was measured between anthocyanins and tyrosinase inhibition (0.740), followed by α-glucosidase (0.737), AChE (0.714), and α-amylase (0.601) inhibitions. These results are consistent with recent papers reporting that flavonoids (mainly anthocyanins, flavonols, and flavones) are one of the most important classes of natural enzyme inhibitors [38,39,40,41,42,43]. Interestingly, negative and significant correlation coefficients were found when considering enzymatic assays and anthocyanins characterizing tuber extracts. In this regard, only two anthocyanins were putatively annotated in tuber extracts, namely Vitisin A and Petunidin 3,5-O-diglucoside, likely characterized by no inhibitory potential. Regarding leaf extracts, no significant correlation coefficients were observed (Supplementary Table S1). Therefore, our findings suggested that a clear matrix-effect mainly due to specific anthocyanins is conceivable, when looking at the correlation coefficients observed. Further works based on in silico studies are strongly required to confirm our findings. 

Regarding the in vitro antioxidant potential, anthocyanins, flavonols, and flavones characterizing *C. triphyllum* flower extracts showed significant correlations with DPPH radical scavenging ability, recording values of 0.974, 0.972, and 0.880, respectively. Looking at the tuber extracts, we found that lignans, anthocyanins, and flavones were the most interesting classes in terms of correlation potential (Supplementary Table S1). In this regard, flavones were strongly (*p* < 0.01) correlated to FRAP (0.882), CUPRAC (0.870), ABTS (0.758), and DPPH (0.744), while lignans recorded the highest correlation coefficient for CUPRAC (0.775). Interestingly, the anthocyanins characterizing tuber extracts showed negative correlations coefficients (*p* < 0.01) with each antioxidant assay (Supplementary Table S1). Overall, it is also important to emphasize the significant correlation coefficients recorded with enzymatic assays, when considering lignans and tyrosols extracted from tubers. Regarding lignans, *C. triphyllum* tubers were mainly characterized by isomeric forms of matairesinol (Supplementary Table S1); in the last years, the interest on lignans has risen because of their estrogenic-like effects but also when considering the potential inhibitory activity on several enzymes, as revealed by in silico and molecular docking studies [44]. Finally, looking at the correlation results for *C. triphyllum* leaf extracts, few significant and positive correlation coefficients were recorded (Supplementary Table S1); overall, the phenolic profile of leaf extracts (in terms of flavonoids and phenolic acids), showed a good degree of correlation with the total in vitro antioxidant capacity (0.01 < *p* < 0.05). Clearly, the observed correlation values between phytochemical profiles (by UHPLC-QTOF mass spectrometry) and antioxidant/biological assays may be explained by considering a different exposure of abiotic and biotic stress factors for each plant part, thus leading to the production of different levels of secondary metabolites. However, looking to the wide diversity in both phenolics and alkaloids of *C. triphyllum* extracts, further studies based on in silico and targeted compound evaluations appear to be worthwhile, to confirm the potential of this plant for food and pharmaceutical purposes. 

## 4. Conclusions

In this work, we focused the attention on the unexplored species *Colchicum triphyllum* Kunze, belonging to the *Colchicum* genus, considering the scarcity of comprehensive studies on its phytochemical profiling and biological properties. Therefore, this study reports the biochemical characterization of different *C. triphyllum* extracts, according to their phenolic and alkaloid compositions, together with the evaluation of their in vitro antioxidant and enzyme inhibitory activities. In this regard, UHPLC-QTOF-MS allowed us to identify 289 compounds (mainly alkaloids and flavonoids). Regarding the plant parts studied, *C. triphyllum* tuber extracts were the most important source of both alkaloids and polyphenolic compounds, being on average 2.89 and 10.41 mg/g, respectively. Multivariate statistics following metabolomics showed a higher impact of the different extraction methods (i.e., maceration, infusion, and Soxhlet) on the polyphenolic profile rather than alkaloids. Overall, *C. triphyllum leaf* extracts showed the stronger in vitro antioxidant capacity (as CUPRAC and FRAP), while each *C. triphyllum* methanolic extract analyzed was active against the enzyme tyrosinase. Strong correlations (*p* < 0.01) were also observed between the phytochemical profiles (mainly lignans and tyrosol equivalents) and the activities determined. Therefore, although characterized by the presence of toxic alkaloids (such as colchicine), this work sustained the utilization of different extraction methods to produce rich extracts in terms of (poly)-phenols and alkaloids, largely contributing to both antioxidant and other pharmacological properties, making *C. triphyllum* a promising source of drugs and whitening agents for both food, pharmaceutical, and cosmetic industries. However, further studies are strongly recommended to understand better the toxicity and bioavailability of the putatively identified phytochemicals, aimed at replacing synthetic antioxidants and enzyme inhibitors.

## Figures and Tables

**Table 1:** Semi-quantitative values for the main phenolic sub-classes and total alkaloids by ultra-high-performance liquid chromatography quadrupole time-of-flight (UHPLC-QTOF) mass spectrometry of the tested extracts together with extraction yields. Values are reported as the mean ± standard deviation (*n* = 3). The results are expressed as mg equivalents (Eq.)/g dry matter. Different superscript letters in the same column indicate significant differences (*p* < 0.05), as determined by Duncan’s post-hoc test. nd = not detected.

Parts	Methods	Extraction Yield (%)	Total Alkaloids (mg Eq./g)	Anthocyanins (mg Eq./g)	Flavones (mg Eq./g)	Flavonols (mg Eq./g)	Phenolic Acids (mg Eq./g)	Lignans (mg Eq./g)	Tyrosols (mg Eq./g)
Flowers	Infusion	35.12	1.49 ± 0.49 ^ab^	0.57 ± 0.05 ^d^	1.98 ± 0.2 ^c^	1.48 ± 0.21 ^c^	0.11 ± 0.02 ^a^	2.27 ± 0.29 ^ab^	0.72 ± 0.35 ^ab^
	Maceration-MeOH	42.6	1.77 ± 0.44 ^abc^	0.78 ± 0.01 ^f^	1.99 ± 0.94 ^c^	1.71 ± 0.20 ^d^	0.20 ± 0.00 ^c^	2.67 ± 1.36 ^abc^	1.09 ± 0.24 ^bc^
	Maceration-Water	26.65	2.08 ± 0.02 ^bcd^	0.06 ± 0.00 ^b^	0.47 ± 0.08 ^a^	0.14 ± 0.00 ^a^	0.19 ± 0.01 ^bc^	3.01 ± 0.28 ^abc^	0.44 ± 0.03 ^a^
	Soxhlet-MeOH	51.25	1.39 ± 0.01 ^ab^	0.67 ± 0.01 ^e^	2.52 ± 0.14 ^c^	1.67 ± 0.01 ^d^	0.15 ± 0.03 ^abc^	1.28 ± 1.30 ^a^	0.42 ± 0.01 ^a^
Average value			1.68	2.08	1.74	1.25	0.16	2.31	0.67
Tubers	Infusion	13.44	1.86 ± 1.00 ^abc^	0.005 ± 0.00 ^a^	0.15 ± 0.00 ^a^	nd ^a^	0.50 ± 0.02 ^g^	4.57 ± 0.03 ^bc^	1.22 ± 0.05 ^cd^
	Maceration-MeOH	28.41	3.19 ± 0.03 ^cd^	nd ^a^	1.06 ± 0.39 ^b^	nd ^a^	0.45 ± 0.05 ^fg^	10.37 ± 0.72 ^d^	1.84 ± 0.05 ^e^
	Maceration-Water	44.02	3.46 ± 0.74 ^d^	nd ^a^	0.39 ± 0.24 ^a^	nd ^a^	0.41 ± 0.01 ^e^	4.84 ± 1.41 ^bc^	1.60 ± 0.01 ^de^
	Soxhlet-MeOH	12.68	3.08 ± 1.14 ^cd^	nd ^a^	0.45 ± 0.02d ^a^	nd ^a^	0.35 ± 0.10 ^de^	10.60 ± 2.50 ^d^	2.85 ± 0.72 ^f^
Average value			2.89	<0.01	0.51	nd	0.43	7.59	1.88
Leaves	Infusion	25.58	2.26 ± 0.49 ^abcd^	0.06 ± 0.00 ^b^	0.33 ± 0.01 ^a^	0.07 ± 0.01 ^a^	0.21 ± 0.01 ^c^	4.77 ± 1.25 ^d^	3.43 ± 0.07 ^g^
	Maceration-MeOH	31.48	1.10 ± 0.54 ^a^	0.05 ± 0.01 ^b^	0.22 ± 0.04 ^a^	0.11 ± 0.00 ^a^	0.30 ± 0.04 ^d^	1.65 ± 0.57 ^a^	0.59 ± 0.09 ^a^
	Maceration-Water	22.88	2.28 ± 0.04 ^abcd^	0.13 ± 0.00 ^c^	1.10 ± 0.01 ^b^	0.43 ± 0.00 ^b^	0.49 ± 0.06 ^fg^	4.99 ± 2.75 ^c^	2.48 ± 0.01 ^f^
	Soxhlet-MeOH	33.61	2.79 ± 1.70 ^bcd^	0.07 ± 0.01 ^b^	0.56 ± 0.00 ^ab^	0.09 ± 0.01 ^a^	0.12 ± 0.00 ^ab^	3.11 ± 0.15 ^abc^	1.45 ± 0.04 ^cde^
Average value			2.11	0.08	0.55	0.17	0.28	3.63	1.87

**Table 2:** In vitro antioxidant activities of the tested extracts. Values are reported as the mean ± S.D. TE: Trolox equivalent; EDTAE: ethylenediaminetetraacetic acid equivalent. Different superscript letters in the same column indicate significant differences (*p* < 0.05), as determined by Duncan’s post-hoc test.

Parts	Methods	Phosphomolybdenum (mmol TE/g)	DPPH (mg TE/g)	ABTS (mg TE/g)	CUPRAC (mg TE/g)	FRAP (mg TE/g)	Metal Chelating (mg EDTAE/g)
Flowers	Infusion	0.51 ± 0.04 ^b^	31.69 ± 0.45 ^c^	27.47 ± 1.57 ^c^	35.00 ± 0.60 ^a^	25.02 ± 0.34 ^a^	20.90 ± 3.06 ^e^
	Maceration-MeOH	0.46 ± 0.07 ^ab^	33.99 ± 0.79 ^cd^	20.53 ± 1.91 ^a^	41.52 ± 1.13 ^c^	25.71 ± 1.78 ^a^	14.40 ± 0.55 ^c^
	Maceration-water	1.10 ± 0.15 ^d^	24.34 ± 0.60 ^b^	19.26 ± 0.64 ^a^	37.00 ± 0.22 ^ab^	38.88 ± 0.67 ^d^	21.53 ± 1.41 ^ef^
	Soxhlet-MeOH	0.74 ± 0.09 ^c^	34.63 ± 0.27 ^c^^d^	24.48 ± 1.15 ^b^	58.31 ± 0.89 ^f^	30.72 ± 0.64 ^b^	16.04 ± 0.41 ^cd^
Average value		0.70	31.16	22.93	42.96	30.08	18.22
Tubers	Infusion	0.33 ± 0.02 ^a^	12.14 ± 10.18 ^a^	30.38 ± 0.38 ^d^	38.38 ± 0.63 ^b^	28.92 ± 0.74 ^b^	33.88 ± 0.39 ^g^
	Maceration-MeOH	0.72 ± 0.02 ^c^	46.17 ± 1.15 ^f^	50.93 ± 0.90 ^h^	63.12 ± 1.52 ^g^	46.16 ± 0.52 ^e^	7.48 ± 0.27 ^b^
	Maceration-water	0.77 ± 0.05 ^c^	29.12 ± 1.05 ^bc^	45.27 ± 0.60 ^f^	44.75 ± 0.32 ^d^	35.34 ± 0.71 ^c^	23.99 ± 1.14 ^f^
	Soxhlet-MeOH	0.85 ± 0.07 ^c^	38.19 ± 0.91 ^de^	38.79 ± 0.31 ^e^	50.65 ± 2.06 ^e^	35.85 ± 0.06 ^c^	1.89 ± 0.25 ^a^
Average value		0.67	31.40	41.34	49.22	36.57	16.81
Leaves	Infusion	1.25 ± 0.09 ^e^	44.91 ± 0.35 ^f^	47.90 ± 0.62 ^g^	68.13 ± 0.45 ^g^	60.66 ± 0.66 ^f^	32.89 ± 2.20 ^g^
	Maceration-MeOH	1.38 ± 0.05 ^e^	45.49 ± 0.74 ^f^	52.55 ± 1.65 ^h^	109.63 ± 2.06 ^l^	70.04 ± 3.09 ^h^	22.26 ± 0.45 ^ef^
	Maceration-water	1.52 ± 0.02 ^f^	42.85 ± 1.09 ^ef^	43.95 ± 1.34 ^f^	84.71 ± 1.74 ^i^	65.92 ± 1.39 ^g^	15.74 ± 2.33 ^cd^
	Soxhlet-MeOH	1.35 ± 0.10 ^e^	47.65 ± 0.38 ^f^	55.73 ± 0.36 ^i^	123.34 ± 2.24 ^m^	70.80 ± 0.88 ^h^	18.25 ± 0.29 ^d^
Average value		1.37	45.22	50.03	96.45	66.67	22.28

**Table 3:** Enzyme inhibitory effects of the tested extracts. Values are reported as the mean ± S.D. Different superscript letters in the same column indicate significant differences (*p* < 0.05), as determined by Duncan’s post-hoc test. GALAE: Galatamine equivalent; KAE: Kojic acid equivalent; ACAE: Acarbose equivalent; nd: not detected.

Parts	Methods	AChE Inhibition (mg GALAE/g)	BChE Inhibition (mg GALAE/g)	Tyrosinase Inhibition (mg KAE/g)	α-amylase Inhibition (mmol ACAE/g)	α-glucosidase Inhibition (mmol ACAE/g)
Flowers	Infusion	nd ^a^	nd ^a^	nd ^a^	0.14 ± 0.01 ^ab^	nd ^a^
	Maceration-MeOH	3.41 ± 0.12 ^d^	nd ^a^	104.09 ± 3.66 ^c^	0.66 ± 0.01 ^ef^	2.69 ± 0.02 ^b^
	Maceration-Water	nd ^a^	nd ^a^	nd ^a^	0.27 ± 0.00 ^c^	nd ^a^
	Soxhlet-MeOH	4.09 ± 0.13 ^f^	nd ^a^	102.52 ± 0.56^c^	0.61 ± 0.06 ^d^	2.73 ± 0.01^c^
Average value		1.87	nd	51.65	0.42	1.35
Tubers	Infusion	nd ^a^	nd ^a^	nd ^a^	0.13 ± 0.00 ^a^	nd ^a^
	Maceration-MeOH	4.80 ± 0.02 ^h^	3.91 ± 0.30^b^	118.70 ± 0.85 ^d^	0.73 ± 0.01 ^g^	nd ^a^
	Maceration-Water	nd ^a^	nd ^a^	nd ^a^	0.18 ± 0.01 ^b^	nd ^a^
	Soxhlet-MeOH	5.11 ± 0.05 ^i^	7.42 ± 1.48 ^c^	118.61 ± 1.41 ^d^	0.64 ± 0.01 ^de^	nd ^a^
Average value		2.48	2.83	59.32	0.42	nd
Leaves	Infusion	2.45 ± 0.16 ^b^	nd ^a^	nd^a^	0.14 ± 0.002 ^ab^	nd ^a^
	Maceration-MeOH	3.62 ± 0.16 ^e^	nd ^a^	125.78 ± 0.13 ^f^	0.71 ± 0.05 ^fg^	2.75 ± 0.01 ^d^
	Maceration-Water	2.95 ± 0.08 ^c^	nd ^a^	4.10 ± 0.34 ^b^	0.24 ± 0.00 ^c^	nd ^a^
	Soxhlet-MeOH	4.56 ± 0.09 ^g^	nd ^a^	121.78 ± 0.57 ^e^	0.73 ± 0.02 ^g^	2.76 ± 0.01 ^d^
Average value		3.39	nd	62.91	0.45	1.37

## Supplementary Materials

The following are available online at https://www.mdpi.com/2304-8158/9/4/457/s1, Table S1: Dataset contained all the putatively annotated compounds by means of UHPLC-QTOF mass spectrometry, together with VIP markers from OPLS-DA and Pearson’s correlation coefficients between phytochemical profiles and biological assays for each plant part.

## References

[1] Toplan G.G., Gurer C., Mat A. (2016). Importance of Colchicum species in modern therapy and its significance in Turkey. J. Fac. Pharm..

[2] Alali F.Q., Tawaha K., El-Elimat T. (2007). Determination of (–)-demecolcine and (–)-colchicine content in selected Jordanian Colchicum species. Int. J. Pharm. Sci. Res..

[3] Terkeltaub R.A., Furst D.E., Bennett K., Kook K.A., Crockett R.S., Davis M.W. (2010). High versus low dosing of oral colchicine for early acute gout flare: Twenty-four-hour outcome of the first multicenter, randomized, double-blind, placebo-controlled, parallel-group, dose-comparison colchicine study. Arthritis Rheum..

[4] Sakane T., Takeno M. (2000). Novel approaches to Behcet’s disease. Expert Opin. Investig. Drugs.

[5] Cifuentes M., Schilling B., Ravindra R., Winter J., Janik M.E. (2006). Synthesis and biological evaluation of B-ring modi ed colchicine and iso colchicine analogs. Bioorg. Med. Chem. Lett..

[6] Cocco G., Chu D.C., Pandolfi S. (2010). Colchicine in Clinical Medicine. A Guide for Internists. Eur. J. Intern. Med..

[7] Ondra P., Válka I., Vičar J., Sütlüpinar N., Šimánek V. (1995). Chromatographic determination of constituents of the genus Colchicum (Liliaceae). J. Chromatogr. A.

[8] Brvar M., Ploj T., Kozelj G., Mozina M., Noc M., Bunc M. (2004). Case report: Fatal poisoning with *Colchicum autumnale*. Crit. Care.

[9] Rocchetti G., Senizza B., Zengin G., Okur M.A., Montesano D., Yildiztugay E., Lobine D., Mahomoodally M.F., Lucini L. (2019). Chemical Profiling and Biological Properties of Extracts from Different Parts of *Colchicum Szovitsii* Subsp. Szovitsii. Antioxidants.

[10] Senizza B., Rocchetti G., Ghisoni S., Busconi M., De Los Mozos Pascual J., Fernandez A., Lucini L., Trevisan M. (2019). Identification of phenolic markers for saffron authenticity and origin: An untargeted metabolomics approach. Food Res. Int..

[11] Mohamed M.B., Rocchetti G., Montesano D., Ali S.B., Guasmi F., Grati-Kamoun N., Lucini L. (2018). Discrimination of Tunisian and Italian extra-virgin olive oils according to their phenolic and sterolic fingerprints. Food Res. Int..

[12] Fellah B., Rocchetti G., Senizza B., Giuberti G., Bannour M., Ferchichi A., Lucini L. (2020). Untargeted metabolomics reveals changes in phenolic profile following *in vitro* large intestine fermentation of non-edible parts of *Punica granatum* L.. Food Res. Int..

[13] Salek R.M., Neumann S., Schober D., Hummel J., Billiau K., Kopka J., Correa E., Reijmers T., Rosato A., Tenori L. (2015). COordination of Standards in MetabOlomicS (COSMOS): Facilitating integrated metabolomics data access. Metabolomics.

[14] Schrimpe-Rutledge A.C., Codreanu S.G., Sherrod S.D., McLean J.A. (2016). Untargeted metabolomics strategies-Challenges and emerging directions. J. Am. Soc. Mass Spectrom..

[15] Zengin G. (2016). A study on *in vitro* enzyme inhibitory properties of *Asphodeline anatolica*: New sources of natural inhibitors for public health problems. Ind. Crops Prod..

[16] Tufa T., Damianakos H., Zengin G., Graikou K., Chinou I. (2019). Antioxidant and enzyme inhibitory activities of disodium rabdosiin isolated from *Alkanna sfikasiana* Tan, Vold and Strid. S. Afr. J. Bot..

[17] Mykhailenko O., Kovalyov V., Goryacha O., Ivanauskas L., Georgiyants V. (2019). Biologically active compounds and pharmacological activities of species of the genus Crocus: A review. Phytochemistry.

[18] Moratalla-López N., Bagur M.J., Lorenzo C., Martínez-Navarro M.E., Salinas M.R., Alonso G.L. (2019). Bioactivity and Bioavailability of the Major Metabolites of Crocus sativus L. Flower. Molecules.

[19] Amin Mir M., Parihar K., Tabasum U., Kumari E. (2016). Estimation of alkaloid, saponin and flavonoid, content in various extracts of *Crocus sativa*. J. Med. Plant Res..

[20] Hosseinzadeh H., Younesi H.M. (2002). Antinociceptive and anti-inflammatory effects of *Crocus sativus* L. stigma and petal extracts in mice. BMC Pharmacol..

[21] Jadouali S.M., Atifi H., Mamouni R., Majourhat K., Bouzoubaâ Z., Laknifli A., Faouzi A. (2019). Chemical characterization and antioxidant compounds of flower parts of Moroccan crocus sativus L.. J. Saudi Soc. Agric. Sci..

[22] Suica-bunghez I., Ion R., Teodorescu I., Sorescu A., Stirbescu R., Stirbescu N. (2017). Fitochemical and antioxidant characterization of autumn crocus (*Colchicum autumnale*) flowers and tubers plant extracts. J. Sci. Arts.

[23] Lapornik B., Prošek M., Golc Wondra A. (2005). Comparison of extracts prepared from plant by-products using different solvents and extraction time. J. Food Eng..

[24] Larsson S., Rønsted N. (2014). Reviewing Colchicaceae Alkaloids – Perspectives of Evolution on Medicinal Chemistry. Curr. Top. Med. Chem..

[25] Borrelli F., Borbone N., Capasso R., Montesano D., De Marino S., Aviello G., Aprea G., Masone S., Izzo A.A. (2009). Potent relaxant effect of a *Celastrus paniculatus* extract in the rat and human ileum. J. Ethnopharmacol..

[26] Montesano D., Rocchetti G., Putnik P., Lucini L. (2018). Bioactive profile of pumpkin: An overview on terpenoids and their health-promoting properties. Curr. Opin. Food Sci..

[27] Rocchetti G., Pellizzoni M., Montesano D., Lucini L. (2018). Italian *Opuntia ficus-indica* cladodes as rich source of bioactive compounds with health-promoting properties. Foods.

[28] Pandey K.B., Rizvi S.I. (2009). Plant polyphenols as dietary antioxidants in human health and disease. Oxid. Med. Cell. Logev..

[29] Saucedo-Pompa S., Torres-Castillo J.A., Castro-López C., Rojas R., Sánchez-Alejo E.J., Ngangyo-Heya M., Martínez-Ávila G.C.G. (2018). Moringa plants: Bioactive compounds and promising applications in food products. Food Res. Int..

[30] de Morais Cardoso L., Pinheiro S.S., Martino H.S.D., Pinheiro-Sant’Ana H.M. (2017). Sorghum (Sorghum bicolor L.): Nutrients, bioactive compounds, and potential impact on human health. Crit. Rev. Food Sci. Nutr..

[31] Borbone N., Borrelli F., Montesano D., Izzo A.A., De Marino S., Capasso R., Zollo F. (2007). Identification of a new sesquiterpene polyol ester from *Celastrus paniculatus*. Planta Med..

[32] Granato D., Shahidi F., Wrolstad R., Kilmartin P., Melton L.D., Hidalgo F.J., Miyashita K., Camp J.V., Alasalvar C., Ismail A.B. (2018). Antioxidant activity, total phenolics and flavonoids contents: Should we ban *in vitro* screening methods?. Food Chem..

[33] Ahmadinejad F., Geir Møller S., Hashemzadeh-Chaleshtori M., Bidkhori G., Jami M.-S. (2017). Molecular mechanisms behind free radical scavengers function against oxidative stress. Antioxidants.

[34] Silman I., Sussman J.L. (2005). Acetylcholinesterase: ‘classical’ and ‘non-classical’ functions and pharmacology. Curr. Opin. Pharmacol..

[35] Zolghadri S., Bahrami A., Hassan Khan M.T., Munoz-Munoz J., Garcia-Molina F., Garcia-Canovas F., Saboury A.A. (2019). A comprehensive review on tyrosinase inhibitors. J. Enzym. Inhib. Med. Chem..

[36] Geromichalos G.D., Lamari F.N., Papandreou M.A., Trafalis D.T., Margarity M., Papageorgiou A., Sinakos Z. (2012). Saffron as a source of novel acetylcholinesterase inhibitors: Molecular docking and *in vitro* enzymatic studies. J. Agric. Food Chem..

[37] Menghini L., Leporini L., Vecchiotti G., Locatelli M., Carradori S., Ferrante C., Zengin G., Recinella L., Chiavaroli A., Leone S. (2018). *Crocus sativus* L. stigmas and byproducts: Qualitative fingerprint, antioxidant potentials and enzyme inhibitory activities. Food Res. Int..

[38] Rocchetti G., Giuberti G., Busconi M., Marocco A., Trevisan M., Lucini L. (2020). Pigmented sorghum polyphenols as potential inhibitors of starch digestibility: An *in vitro* study combining starch digestion and untargeted metabolomics. Food Chem..

[39] Rocchetti G., Giuberti G., Gallo A., Bernardi J., Marocco A., Lucini L. (2018). Effect of dietary polyphenols on the *in vitro* starch digestibility of pigmented maize varieties under cooking conditions. Food Res. Int..

[40] Tan Y., Chang S.K.C. (2017). Digestive enzyme inhibition activity of the phenolic substances in selected fruits, vegetables and tea as compared to black legumes. J. Funct. Foods.

[41] Martinez-Gonzalez A.I., Díaz-Sánchez Á.G., de la Rosa L.A., Bustos-Jaimes I., Alvarez-Parrilla E. (2019). Inhibition of α-amylase by flavonoids: Structure activity relationship (SAR). Spectrochim. Acta A.

[42] Xiao J., Kai G., Yamamoto K., Chen X. (2013). Advance in Dietary Polyphenols as α-Glucosidases Inhibitors: A Review on Structure-Activity Relationship Aspect. Crit. Rev. Food Sci. Nutr..

[43] Takahama U., Hirota S. (2018). Interactions of flavonoids with α-amylase and starch slowing down its digestion. Food Funct..

[44] Mocan A., Zengin G., Crisan G., Mollica A. (2016). Enzymatic assays and molecular modeling studies of *Schisandra chinensis* lignans and phenolics from fruit and leaf extracts. J. Enzym. Inhib. Med. Chem..

